# Implementation of a clinical pathway may improve alcohol treatment outcome

**DOI:** 10.1186/s13722-015-0031-8

**Published:** 2015-03-07

**Authors:** Anette Søgaard Nielsen, Bent Nielsen

**Affiliations:** Department of Psychiatry, Odense University Hospital, DK-5000 Odense C, Denmark; Unit of Clinical Alcohol Research, Clinical Institute, University of Southern Denmark, DK-5000 Odense C, Denmark

**Keywords:** Alcoholism, Outpatient clinics, Clinical pathway, Local monitoring, Outcome

## Abstract

This article describes the design, implementation, and evaluation of a clinical pathway system in a two-cohort quasi-experimental study before and after implementation, controlling for confounders. The main outcome measures were retention in care and sensible alcohol use (defined as abstinent or drinking no more than 21 standard drinks per week). Patients with harmful alcohol use or dependence as their primary problem who were seeking psychosocial treatment at one of four alcohol clinics in Denmark participated in the study. After implementation of the clinical pathway system, which incorporated a structured intake, a referral and independent follow-up system, checklists, audit, and feedback, there was no change in length of stay, but significantly more patients had a good clinical outcome (stopped or moderated their consumption) at the end of treatment (OR = 1.9; 1.2–3.1). The study documents the feasibility of using a clinical pathway framework, incorporating a local monitoring system, checklists, audit, and feedback to enhance treatment quality and improve outcomes for alcohol use disorders.

## Introduction

The quality of treatment for alcohol use disorders varies widely [1,2], and how treatment quality might be improved and monitored has been a subject of discussion for decades [3]. In 2006, the Institute of Medicine recommended the introduction of substance use treatment systems to strengthen infrastructure and quality assurance through using valid and reliable patient-assessment instruments, building the evidence base for effective treatment methods, and continuously monitoring treatment processes and outcomes [4]. Clinical pathways—that is, structured systems for standardizing care—have the potential to reduce variability in treatment quality and improve systematic data collection for continuous quality improvement [5,6].

In many countries, outpatient psychosocial treatment is the primary vehicle for delivering services for alcohol problems. The treatment is often delivered by relatively small treatment facilities staffed by social workers and therapists. Some health-care areas have a tradition for the development of clinical pathways. Within the medical field, for instance, there is a tradition for the systematic collection of patient data, the delivery of evidence-based treatment, and systematic quality development. This tradition is not present to the same degree in the alcohol treatment field, at least not in Denmark. Although staff working in the alcohol field in Denmark are generally well trained, treatment is often delivered as individual treatment courses by a single therapist behind “a closed door,” even when the treatment facility has a number of staff. The documentation of therapy falls to the therapist and is often inconsistent, lacking a standardized approach, and hinging on what the therapist considers is important to document. Hence, it is not always easy to know what is actually going on during treatment and to what extent specific elements of treatment feature in the treatment course. The implementation of new strategies and routines may also be a challenge and are even more difficult to assess.

From a quality improvement perspective, small clinics delivering treatment for alcohol problems would be considered microsystems. However, when clinics work together in an organized system, it becomes possible to implement clinical pathways and evaluate the impact of specific system interventions. This article describes the development, implementation, and evaluation of a clinical pathway within a Danish alcohol treatment institution consisting of four community-based outpatient clinics with roughly 50 staff members in total.

## Research methods

### The participating clinics

The present study was conducted in the county of Funen, Denmark. A public health organization consisting of four outpatient alcohol treatment clinics delivered services to the whole county, whose population is about 480,000 (about 10% of the Danish population). The four clinics were scattered across the county. Most patients sought treatment at the nearest clinic. In all the clinics, treatment was carried out by an interdisciplinary team of nurses, psychiatrists, and social workers. The treatment options at the clinics were in principle the same. In all four clinics, the patients were offered initial detoxification as needed, and thereafter assessed by a structured interview. The backbone of the interview was the Addiction Severity Index (ASI) [7]. Patients were also asked how many days in the past 30 days they had consumed more than three standard units of alcohol. All data from the interviews were stored in a clinical database. After reviewing the ASI data, the psychiatrists referred the patients to one of the clinic’s treatment interventions. Treatment staff were well trained, delivering the preferred treatment approach in which they had been trained. Staff practice was closely monitored. Since the clinics were all part of the same organization, they shared management, and staff covered for each other in case of illness and vacation. The present study focused not on the specific content of treatment but rather on the systemic aspects of service delivery.

### Development of a clinical pathway

A year-long monitoring program of all treatment courses at the clinics revealed two common quality problems across the clinics: patient dropout from treatment was generally high during the first 6 months of treatment, and relatively few clients reduced their drinking to below the Danish recommended level for sensible drinking (maximum of 21 standard drinks per week) during the treatment course.

The quality problems were presented to staff and management, and a quality improvement unit within the treatment system was set up and run by an audit team. The audit team consisted of management, staff representatives from all participating clinics, and the psychiatrist. Three staff working groups were also set up. All the clinics were represented in each working group in order to secure homogeneity across clinics. Staff and management identified areas that needed to be developed. Within these areas, the working groups were to identify and describe key process standards that they believed could improve patient retention and outcomes. In order to ensure that the staff could talk freely and frankly, and to secure local ownership of the process and the proposals developed, management was not part of the working groups. The working groups met approximately five times during the following 6 months. All meetings were half day-meetings.

**Figure 1:**
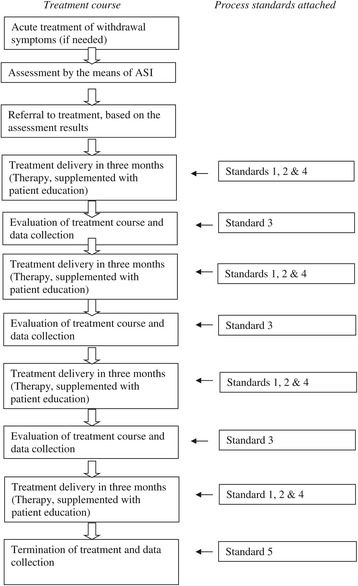
**Clinical pathway in the alcohol treatment facilities in funen.**

The process standards developed by the working groups were presented to the entire staff and management at the clinics during a full-day meeting, and a clinical pathway was designed based on the proposals and identified service-specific timelines. The clinical pathway, with five process standards attached, is shown in Figure 1. All staff agreed to be faithful to the structure and content of the pathway. The audit team oversaw implementation of the standards.

### Process standards

The content of the process standards was based on published research, and the core performance standards developed by the Washington Circle Group [8,9] were particularly taken into account. In all, five process standards were developed by the working groups. They are briefly described below.

### Standard 1: Documentation of therapist adherence to clinical guidelines

The clinics had developed clinical guidelines for the delivery of cognitive-behavioral therapy, systemic family therapy, and supportive counseling. Knowing that therapists often fail to adhere to such clinical guidelines [10], one process standard further required that use of the guidelines should be documented in a standardized way in the case notes for all therapy sessions.

### Standard 2: Patient education

In contemporary alcohol treatment, patients are expected to make their own informed choices and take responsibility for changing their drinking habits. Patient education during treatment ensures that patients have the information they need in order to make the necessary choices [8]. Consequently, a standard for patient education was established. The aim of the standard was to ensure that patient education was delivered when needed. The standard described how the staff should deliver information about how alcohol affects people, the risk of incurring an alcohol use disorder, and what the symptoms of dependency are. The patient education material also described alcohol’s possible damage to health, social life, and to the children in a family. The standard specified how the information should be given in a nonthreatening way and be made as personalized as possible.

### Standard 3: continuity of care

To ensure that the treatment course was on the right track, regular assessment, feedback to the patient, and discussion of care should occur. This standard stated that at least once every 3 months, the therapist should present a status report at the staff meeting, based on an ASI interview previously conducted with the patient. Treatment course and progress would then be discussed with the patient and with colleagues.

### Standard 4: retention in care

Clinical research generally shows that retention in treatment is associated with better client outcomes and that the prognosis is particularly good with patients who remain in treatment for 6 months [11]. This standard described strategies to increase retention (e.g., reduction of waiting time early in treatment) and specified strategies for contacting patients who missed appointments (e.g., phone calls and letters) [12].

### Standard 5: termination

A satisfactory conclusion of treatment also seems to be vital [13]. Standard 5 describes how the patient and therapist should decide together when it is time to end treatment. Ideally, it is the patient who initiates termination, but if this does not happen and the patient has remained sober for a reasonable period of time, it is the therapist’s responsibility to bring up the topic of termination.

The working groups described all five standards in the following terms: process, structure, and expected outcome. Each standard described *what* was supposed to be carried out (process); by *whom, when, and where* (structure); and what *result* was intended (outcome). Furthermore, guidance notes were drawn up by the working groups, describing the rationale and the evidence base for the standards, which were distributed to colleagues.

### Process standards checklist

When these process standards were implemented, the audit team introduced a checklist to be completed for each patient to document whether and when the standards had been carried out. The use of such checklists is an important implementation tool and data source in clinical pathways [14]. The checklist also served as a reminder to minimize lapses in standards during the course of treatment. Data from the checklist allowed performance of good practice standards to be linked to client outcomes.

### Evaluating the clinical pathway

Since national monitoring programs were unavailable, we opted for a local outcome monitoring system in which data were collected during treatment [15]. The monitoring system was in place both before and after implementation of the clinical pathway. The data in the monitoring system consisted of details from ASI interviews completed with all patients at intake and at 3, 6, and 12 months after treatment initiation, or at treatment termination. The data yielded a multidimensional assessment of the patient’s functional capacity in the preceding month. ASI ratings were derived for seven areas of functioning: medical, employment, alcohol, drug, legal, family/social network, and psychiatric health, as well as composite scores. In addition to the ASI, we recorded the following data from the case notes: dates of key events (intake, referral, retention in treatment, treatment sessions, treatment length, and termination) and documentation of the use of clinical treatment guidelines. All data were entered in a clinical information system to support reporting and decisionmaking, and were analyzed via SPSS software.

The audit team reviewed retention and outcome data and random treatment courses qualitatively every 6 months. The audit team could decide whether or not new standards should be developed and implemented by other working groups. The present study describes the evaluation of the first five standards.

### Patients

The study population included two consecutive cohorts of patients entering psychosocial treatment at the four alcohol outpatient clinics during either a 6-month pre-pathway period (Cohort one) or a 6-month post-pathway period (Cohort two). All patients were included in the study except those who: were under 18 years old, had severe psychosis or cognitive impairment, did not have a primary alcohol problem, or were not of Danish origin. Data from patients who began treatment more than once during the periods of data collection were only analyzed in the first collection period. The two samples consisted of 228 patients in Cohort one and 309 patients in Cohort two.

### Data analyses

Student’s t-test was used for comparing the mean values of the two cohorts, and Chi2 test was employed for bivariate data.

In order to follow potential developments in the level of implementation of the standards, the post-pathway period was divided into three time periods before analysis, each of 2 months’ duration. The analysis focused on a) retention in care; and b) sensible consumption at treatment termination. The reference group included patients entering during the pre-pathway period (Cohort one). Logistic regression models were used to control for severity of alcohol abuse at initiation of treatment (confounder). The odds ratio was given with 95 percent confidence limit.

The analysis of outcome was based on intention-to-treat, irrespective of whether the patients had completed treatment or not. Although not without limitations [16], last observation carried forward (LOCF) was used to address missing data [17]. As level of significance for all tests, alpha = 0.05 was chosen.

## Results

Table 1 shows pre-treatment characteristics and treatment courses for both cohorts. Of four sociodemographic and seven ASI variables at baseline, the two cohorts differed on only one: patients in Cohort two had significantly more severe alcohol problems at baseline (p = 0.002). There was no significant difference in treatment courses between the cohorts.

**Table 1:** **Pre-treatment characteristics and treatment courses of patients referred to outpatient psychosocial treatment before and after implementation of the clinical pathway (CP)**

**Variables**	**Cohort 1 (before CP) (N = 228)**	**Cohort 2 (after CP) (N = 309)**	**p values**
**Sociodemographic**			
Female (%)	27.6	29.4	0.645
Age (mean)	43.2 (10.5)	44.1 (10.7)	0.364
Education in yrs (mean)	9.6 (1.5)	9.6 (1.5)	0.818
Currently cohabiting (%)	46.9	49.2	0.604
**ICD - diagnostic**			
F10.1. Harmful use (%)	16.2	13.2	0.459
F10.2. Dependence syndrome (%)	83.8	86.8	
**ASI problem scores** ^**a**^ **(mean)**			
Medical	0.317 (0.336)	0.321 (0.351)	0.954
Employment	0.557 (0.290)	0.540 (0.302)	0.496
Alcohol use	0.538 (0.235)	0.604 (0.238)	0.002
Drug use	0.015 (0.050)	0.012 (0.046)	0.052
Legal status	0.032 (0.100)	0.028 (0.083	0.978
Family/social	0.220 (0.221)	0.217 (0.236)	0.453
Psychiatric	0.226 (0.227)	0.219 (0.219)	0.685
**Treatment course**			
Treatment sessions (mean)	6.6 (5.3)	6.1 (4.7)	0.234
Treatment length in days (mean)	149 (125.6)	144 (121.9)	0.612
Retention in treatment for 6 months (%)	47	40	0.326

^a^Based on Addiction Severity Index – composite score. Scores vary from 0 (no problem) to 1 (extreme problem) in preceding 30 days.

How well staff adhere to the process standards is crucial to a quality improvement project like the present one. Table 2 reports adherence to the five process guidelines in the three 2-month intervals of the post-pathway period. For Standards 1–4, average adherence was 58 percent and varied across time periods. The data describing adherence to Standard 5 showed how only 24 percent of patients completed a planned termination of treatment with the therapist. The only standard to which staff adherence improved significantly during the 6-month post-pathway period was Standard 3 (continuity of care), which improved from 58 percent to 72 percent, a 24-percent increase; p = 0.03.

**Table 2:** **Adherence with process standards**

**Process standards**	**(Share of patients where standards were followed)**		
	**0-2 months after implementation of CP (N = 107) %**	**3-4 months after implementation of CP (N = 104) %**	**5-6 months after implementation of CP (N = 98) %**
Standard 1: Percent of patients where therapist’s adherence to the clinical guidelines has been documented.	55	59	64
Standard 2: Percent of patients who are advised or given information about alcohol disorders.	55	48	53
Standard 3: Percent of patients discussed at team meetings every 3 months (continuity of care).	58	50	72
Standard 4: Percent of patients where therapist adheres to standards for retention in care.	64	57	63
Standard 5: Percent of patients who complete the phase of termination with the therapist.	30	18	24

**Figure 2:**
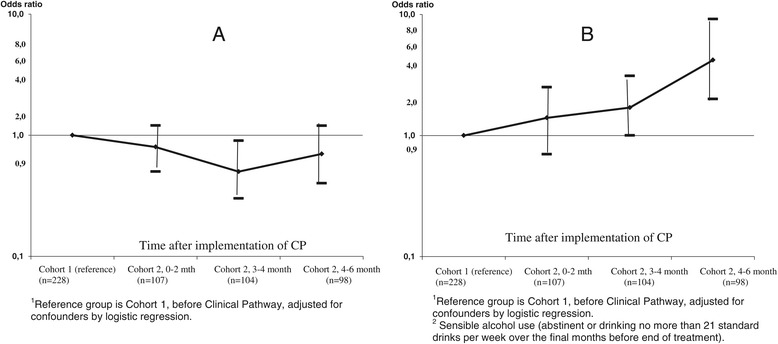
**Changes in outcome indicators concerning retention and sensible alcohol use in patients referred to individual psychosocial treatment. 2A** Odds ratio^1^ (95% CL) for retention in care during the first 6 months of psychosocial treatment. **2B** Odds ratio^1^ (95% CL) for good clinical outcome^2^ (LOCF).

Despite modest staff adherence and no overall change in retention, patients in Cohort two were substantially more likely to have a good clinical outcome [see Figure 2; OR = 1.9 (1.2–3.1)], with a pattern of improvement throughout post-implementation monitoring. Patients in Cohort two also showed significantly higher decreases in alcohol problem severity on the ASI during treatment (see Table 3). Furthermore, patients with a good clinical outcome in terms of sensible consumption also showed fewer problems in the areas of alcohol, drug, family/social network, and psychiatric health at the end of treatment than those who did not, measured by the means of ASI composite scores (not shown in the tables).

**Table 3:** **Change from baseline in Addiction Severity Index (ASI) composite scores and self-reported drinking among patients treated before and after implementation of the clinical pathway (CP)** ^**1**^ **intention-to-treat-analyses (LOCF)**

	**Cohort 1 (before CP) N = 228**	**Cohort 2 (after CP) N = 309**	**Difference** ^**2**^	**P-values**
**ASI-composite scores** ^**3**^				
•Medical	−0.030 (0,256)	+0.007 (0.291)	+0.023	0.116
•Employment	−0.021 (0.165)	+0.006 (0.163)	+0.015	0.058
•Alcohol use	−0.223 (0.261)	−0.274 (0.280)	−0.051	0.029
•Drug use	+0.010 (0.211)	−0.005 (0.037)	−0.015	0.217
•Legal status	−0.014 (0.079)	−0.018 (0.080)	−0.004	0.567
•Family/social	−0.047 (0.196)	−0.048 (0.187)	−0.001	0.930
•Psychiatric	−0.006 (0.218)	−0.007 (0.184)	−0.002	0.538
**Self-reported drinking** ^**4**^				
•Abstinent (days)	+5.0 (11.6)	+9.0 (11.6)	+4.0	0.001
•< 4 drinks per day	+6.9 (10.4)	+9.3 (71.6)	+2.4	0.012
•Sensible consumption (%)	+31.6	+41.1	+9.5	0.024

^1^Values are mean (SD).

^2^Difference between groups, 12 months after initiation of treatment.

^3^Scores vary from 0 (no-problem) to 1 (extreme problem) in the preceding 30 days.

^4^Based on ASI the preceding 30 days.

## Discussion

Several studies have found how the use of clinical guidelines improves treatment effectiveness and enhances the implementation of evidence-based methods [18], and that continuity in treatment is linked to the likelihood of good outcomes [19]. In a post-only evaluation, the Network for the Improvement of Addiction Treatment demonstrated that process improvement strategies can contribute to enhanced quality of care for alcohol disorders [20], including an increase in patient compliance [21]. We did not find an improvement in retention, but observed a significant increase in good clinical outcomes and a reduction in alcohol problems after the implementation of a clinical pathway.

It is a clear limitation to this study that we do not know to what extent the elements of the standards were met before the clinical pathway was implemented. We know that implementation of the clinical pathway was not entirely successful, in that none of the process standards were met more than 72 percent of the time. There is much room for improvement in adherence to the practice standards, but since a strong focus was placed on implementation during the post-pathway period, the findings might reflect the low level of implementation of the various elements in the pre-pathway period. Difficulties in implementing strategies are well known and are described in studies of clinical pathways in the field of psychiatry. Barriers to successful implementation may include lack of awareness, lack of agreement, little expectancy of improved outcome, and inertia [22,23]. Smaller institutions may also need more time before improvement strategies show effect [21], as demonstrated in other studies in similar areas [24,25]. In other words, results from the implementation of clinical pathways in alcohol treatment appear to be as divergent as those in other health services [26].

Clearly, there are further limitations to this study. We used the principles of intention-to-treat and, in consequence, included all consecutive patients who started treatment. We also used the LOCF method; hence, patients who provided no further data at follow-up were assigned their pre-test data. Thus, patients who dropped out of treatment during the first 3 months had their baseline data allocated as outcome status; patients who dropped out of treatment before the 6-month status had the data obtained at the 3-month status interviews allocated as outcome data, and so on. The LOCF method is, however, not without problems either, and may lead to biased results [16]. Furthermore, the quasi-experimental design relied on a historical control group (Cohort one), with 12 months intervening between the samples. Thus, it is possible that changes other than the implementation of the clinical pathway may have accounted for the improvement in client outcomes during the interval.

Within these limitations, this study offers further support for the effectiveness of a clinical pathway in improving client outcomes. We believe that the local introduction of a clinical pathway can be an effective strategy for microsystems, such as small alcohol treatment clinics to standardize their processes and reduce clinical performance variations. The process of having an audit team continuously overseeing data on retention and outcome and, when necessary, initiating staff working groups to adjust old standards or develop new ones in the treatment course may be a route to securing local ownership of the process. In fact, it has for several years now been a routine practice in the treatment organization under study and serves as a strategy to open “the closed doors” of the therapists. It is not only considered to have improved the outcome of treatment, it is also deemed to have made the treatment much more transparent and of more uniform quality across the clinics.
